# The deformation characteristics and the prefabricated crack pressure relief stability control of a small coal pillar roadway under stress superposition

**DOI:** 10.1038/s41598-026-44430-7

**Published:** 2026-03-27

**Authors:** Shixing Cheng, Zhanguo Ma, Yue Li, Xiao Zhang

**Affiliations:** 1 School of General Education, Shanxi Institute of Science and Technology, Jincheng, 048000 China; 2 School of Mechanics and Civil Engineering, China University of Mining and Technology, Xuzhou, 221116 China

**Keywords:** Small coal pillar roadway, Stress superposition, Energy accumulation, Deformation characteristics, Prefabricated crack, Overlying rock fracture, Energy science and technology, Engineering, Solid Earth sciences

## Abstract

The substantial pressure acting on small coal pillars poses a formidable challenge to maintaining stability during mining operations. Small coal pillar mining under high-stress conditions has thus emerged as one of the most critical bottlenecks in sustainable coal production. In this study, the deformation characteristics of a small coal pillar roadway under stress superposition were investigated via numerical simulation. Physical modeling was further employed to elucidate the impact of prefabricated roof crack on the migration behavior of overlying strata, alongside the development of a pressure-relief stability control strategy. The vertical stress on the small coal pillars was exacerbated by four dynamic pressure events, leading to a surge in energy density, and further exacerbating the energy levels of the small coal pillar. The prefabricated cracks effectively altered the strata caving characteristics and roof overhang structure, the caving angle increased from 55° to 70.5°, and the length of the roof cantilever structure was reduced by 48%. Field applications demonstrated that this prefabricated crack technique achieved remarkable pressure-relief effects, with the vertical stress increment at a depth of 3 m in coal pillar decreasing significantly from 5.5 MPa to 2.5 MPa. These findings provide a robust theoretical and technical foundation for the stability control of high-stress small coal pillar mining panel.

## Introduction

**Fig. 1:**
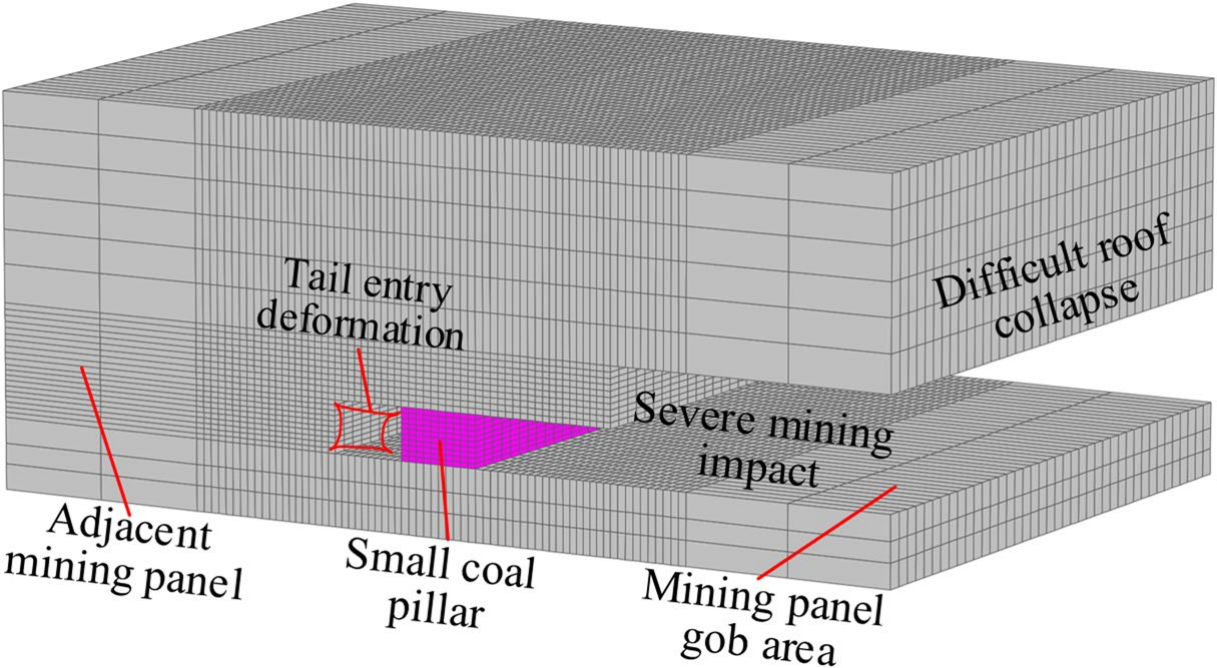
Schematic diagram of a small coal pillar retaining roadway.

The efficient mining of coal, a non-renewable energy source, is essential for economic development^1–4^. Small coal pillar mining has received a lot of attention due to its ability to increase the recovery rate of coal resources, facilitate excavation and mining, and meet the demand for high-intensity mining^5,6^. Still, under the influence of mining, the pressure on small coal pillars^7,8^ is large, and it is difficult to control the stability of small coal pillar roadways^9,10^, as shown in Fig. 1. Small coal pillar mining under high-stress conditions has become one of the most pressing hotspots and difficulties in green coal mining.

In a small coal pillar mining panel, the main roof of the mining panel gob rotates laterally after the panel retreats, and the load imposed by the main roof causes a continuous increase in the deformation of the small pillar roadway^11^. Scholars have studied the failure laws of the surrounding rocks of small coal pillar roadways, as well as the theory and techniques for the stability control of small coal pillars. In terms of the surrounding rock deformation law of small coal pillar roadways, the results of theoretical analyses^12^, numerical calculations^13^, physical simulations^14^, and on-site applications are combined to obtain a reasonable coal pillar size^15–17^. The influence of the coal pillar size on the surrounding rock stress distribution^18^, plastic zone development^19^, and deformation during roadway excavation and the mining panel retreat^20^ on both sides of the small coal pillar are studied, and the surrounding rock deformation mechanism of the small coal pillar^21,22^ roadway is revealed. Current research on the stability of small coal pillar has been extensively explored from various perspectives. These include the creep failure behavior of coal pillar under high-stress conditions^23^, damage identification methods utilizing distributed optical fiber sensing^24^, fracture network evolution in supercritical environments^25^^26^;, and anisotropic failure characteristics under varying loading paths^27^. These investigations offer valuable insights for further elucidating the instability mechanisms of small coal pillar. In terms of the supporting theory and technology of small coal pillar roadways, the asymmetric deformation characteristics of small coal pillar roadways^28^ are discussed. Depending on the failure characteristics of different plastic zones, control methods such as high-strength and high-preload bolt cable support^29^, grouting reinforcement^30,31^, and opposite anchor cable^32^ support have been proposed to increase the bearing capacity^33^ of the surrounding rock of small coal pillar roadways and reduce roadway deformation. Nevertheless, systematic analyses of the stability of small coal pillar throughout their entire service life remain limited, and the failure process from the perspective of energy evolution has yet to be thoroughly investigated.

As the mining intensity increases^34^, it is difficult to meet the requirements for safe and efficient mining by strengthening the support of small coal pillar roadways. Some scholars have proposed pressure relief technology^35,36^ for directional pre-splitting blasting with a deep hole. Directional pre-splitting blasting prefabricates cracks^37,38^ in the roof, which maintains the roof caving in time and reduces the effect of the gob side disturbance stress^39^ on the small coal pillar. The pressure relief mechanisms of prefabricated cracks have been revealed by using numerical simulations to study their effects on the stress and deformation of the small coal pillar roadway surrounding rock^40,41^. Physical simulation serves as a key method for revealing the fracture behavior of overlying strata^42^. Previous research has extensively employed this approach to monitor and analyze displacement fields^43^, fracture evolution^44^, and stress distribution^45^ during the mining of a working face. However, most existing findings are limited to conventional mining conditions^46^. In the context of pressure relief through prefabricated fractures in the roof of small coal pillar, the instability and collapse behavior of overlying strata under the coupled influence of prefabricated fractures and stress redistribution in small pillar roadways have not been sufficiently investigated.

Based on the analysis above, further in-depth research is needed to examine both the long-term stability of small coal pillar roadways and the movement patterns of overlying strata following pressure relief via prefabricated roof fractures. This study takes a small coal pillar roadway as the research object. By using numerical calculations, physical simulation tests, and field applications, the stress superposition, energy accumulation, and the surrounding rock deformation of the small coal pillar roadway were analyzed, and the mining panel overlying rock caving characteristics with a prefabricated crack in the roof were obtained. The study presented in this paper provides a basis for the design of mining replacement, surrounding rock control, and roof management of high-stress small coal pillar mining panels, and it is of great theoretical and practical significance for the maintenance improvement of small coal pillar roadways.

## Stability analysis of the surrounding rock of a small coal pillar roadway with stress superposition

The stability of the roadway on either side of a small coal pillar when it is used to protect the roadway is of great importance for the mining of the two working faces. Thus, analyses of the stress stacking, energy accumulation, and deformation laws of the surrounding rock during excavation and mining on both sides of a small coal pillar reveals the influence of the stress stacking laws on the stability of the small coal pillar roadway’s surrounding rock.

### Numerical calculation model establishment and scheme design

**Fig. 2:**
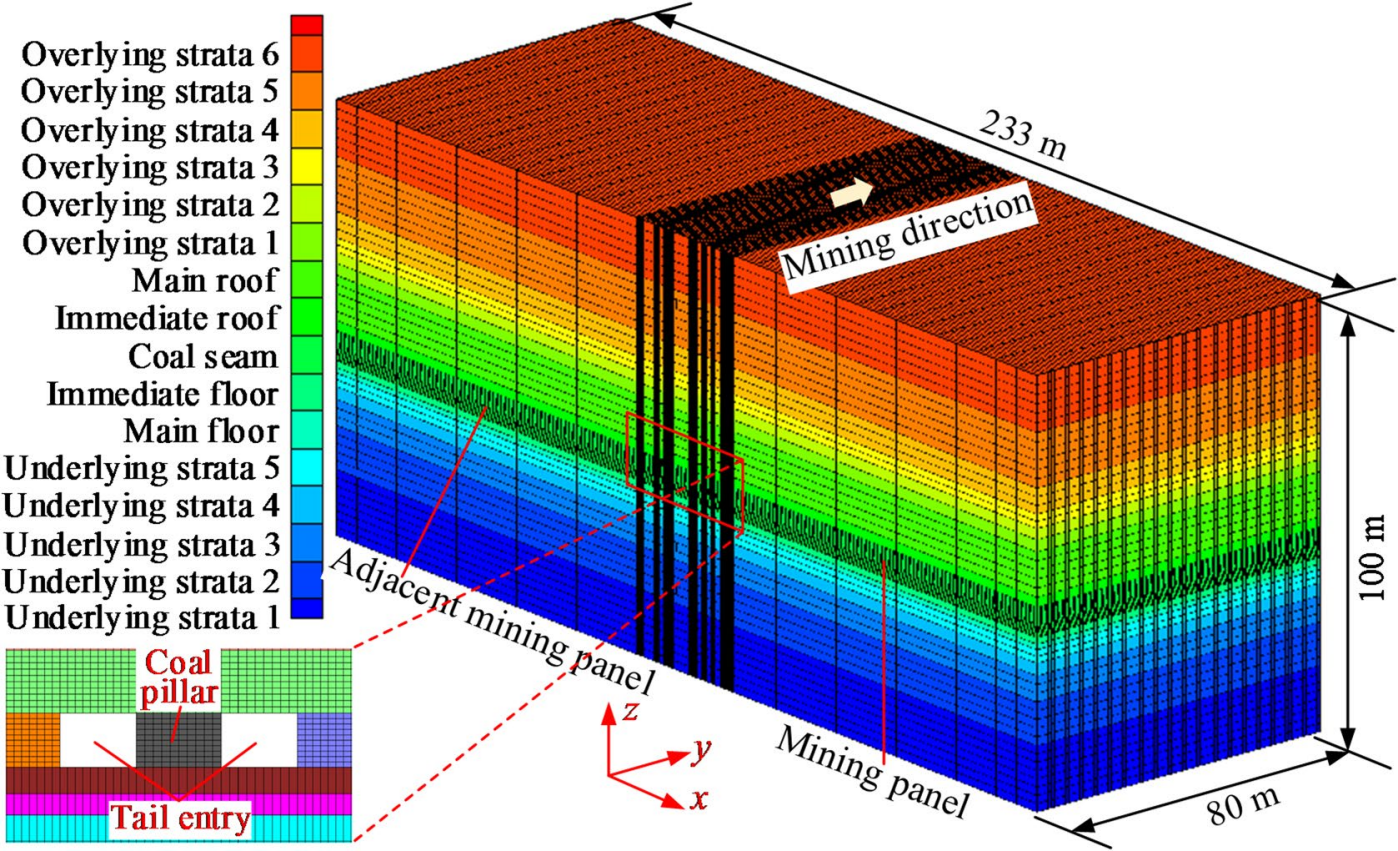
Numerical calculation model.

A FLAC3D numerical calculation model of small coal pillar roadway mining panels was established, as shown in Fig. 2. The length × width × height of the model was 233 m×80 m×100 m. In terms of the coordinate arrangement, the interval of x∈ (0.0,109.5) was the adjacent mining panel, the interval of x∈ (109.5, 114.0) was the adjacent panel gateway, the interval of x∈ (114.0,119.0) was the small coal pillar, the interval of x∈ (119.0,123.5) was the panel gateway, and the interval of x∈ (123.5, 233.0) was the mining panel. The model had a sliding boundary around it and a fixed boundary at the bottom. This model only considered weight stress and neglected the effect of tectonic stress. The equivalent load of the overlying strata was applied to the top of the model. The equivalent load was 8.0 MPa. The model approximated coal and rock masses as homogeneous, continuous, and isotropic media, and it adopted the Mohr–Coulomb yield criterion. The detailed mechanical parameters of coal and rock strata used in the numerical model are shown in Table 1.

After the initial equilibration of the model, the mining panel and the adjacent mining panel gateway were excavated sequentially, and then mining on both sides of the working face was simulated successively to investigate the effect of mining on the stability of the surrounding rock in the roadway. The stress stacking, energy accumulation, and deformation properties of the surrounding rock were analyzed during the excavation and mining of the working faces on either side of the small coal pillar.

**Table 1:** Mechanical parameters of coal and rock strata.

Rock Strata	Density (kg/m^3^)	Friction Angle (°)	Cohesion (MPa)	Shear Modulus (GPa)	Tensile Strength (MPa)	Bulk Modulus (GPa)
Overlying Strata 6	2420	33	1.89	1.53	0.89	2.36
Overlying Strata 5	2250	33	1.56	1.28	0.59	2.78
Overlying Strata 4	2420	33	1.89	1.53	0.89	2.36
Overlying Strata 3	2250	33	1.56	1.28	0.59	2.78
Overlying Strata 2	2595	35	4.64	1.66	1.43	8.62
Overlying Strata 1	2360	32	1.50	1.25	0.53	2.08
Main Roof	2595	35	4.64	1.66	1.43	8.62
Immediate Roof	2420	33	1.89	1.53	0.89	2.36
Coal Seam	1400	28	1.56	1.18	0.58	2.37
Immediate Floor	2250	33	1.56	1.28	0.59	2.78
Main Floor	2695	36	3.45	1.51	1.16	7.62
Underlying Strata 5	2250	33	1.56	1.28	0.59	2.78
Underlying Strata 4	2420	33	1.89	1.53	0.89	2.36
Underlying Strata 3	2695	36	3.45	1.51	1.16	7.62
Underlying Strata 2	2250	33	1.56	1.28	0.59	2.78
Underlying Strata 1	2420	33	1.89	1.53	0.89	2.36

### The superposition effect of surrounding rock stress on the small coal pillar roadway

**Fig. 3:**
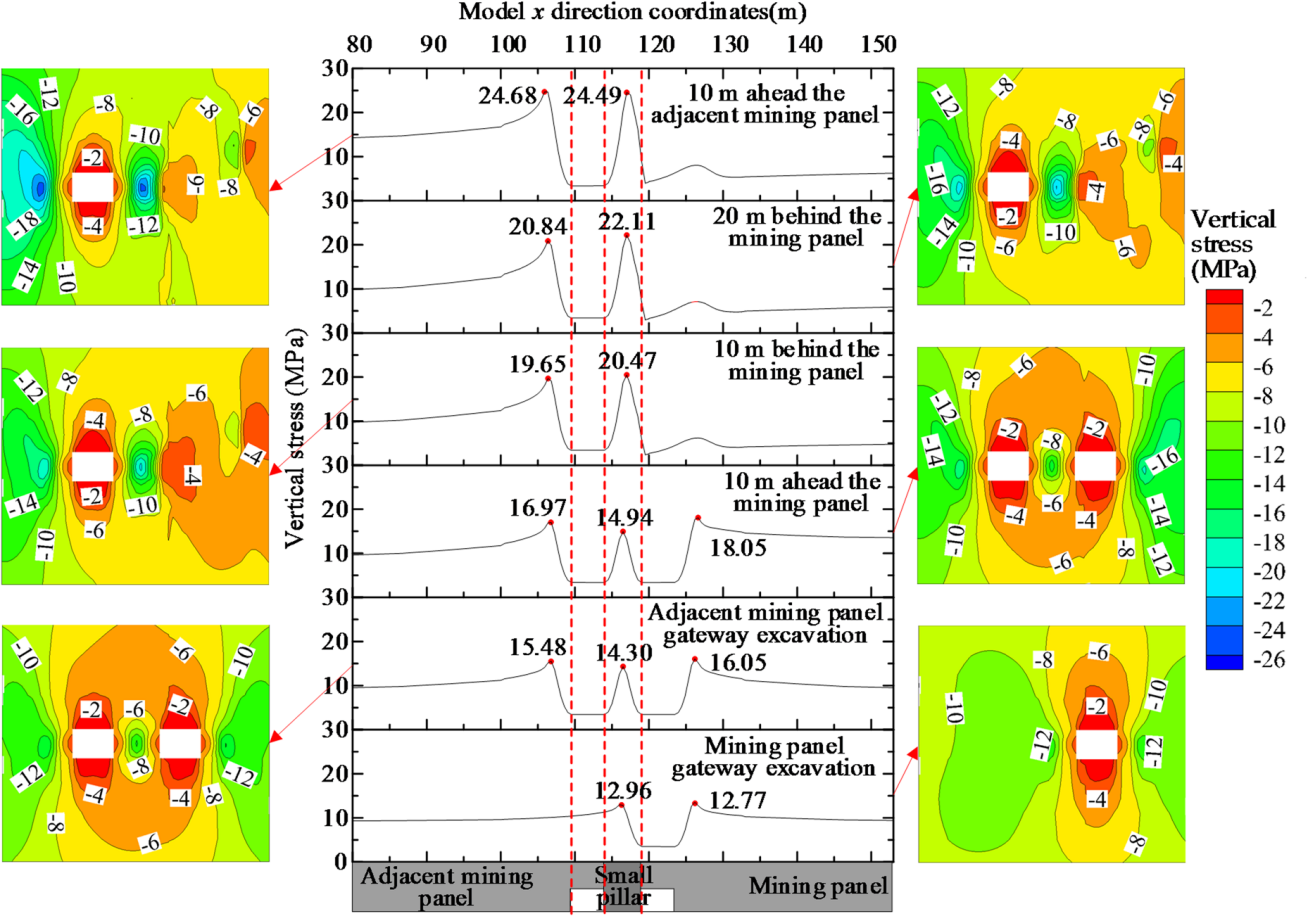
The superposition evolution process of surrounding rock stress in small coal pillar roadway.

The superposition effect of the surrounding rock stresses was further revealed by analyzing the vertical stresses in the small coal pillar roadway during excavation and mining on either side of the pillar. The evolution of the stress superposition in the small coal pillar roadway is shown in Fig. 3.

The vertical stress on the right side of the roadway peaked at 12.77 MPa after excavating the mining panel gateway. The peak value of the vertical stress on the right side increased to 16.05 MPa, a 25.69% increase due to the excavating pressure of the adjacent mining panel gateway. The peak value of the vertical stress on the right side increased to 18.05 MPa at 10 m in front of the mining panel, a 41.35% increase, during the advance of the mining panel. According to the vertical stress nephogram, the vertical stress on the right-side region of the mining panel gateway increased during three different phases: the excavation of the mining panel gateway, the excavation of the adjacent mining panel gateway, and the advance of the mining panel. As a result, during the excavation phase of the adjacent mining panel gateway and the advanced phase of the mining panel, the vertical stress stacking on the right side of the mining panel gateway increased because of the excavation and mining dynamic pressure.

The vertical stress peaked at 12.96 MPa in the area where the small coal pillar was located after the excavation of the mining panel gateway. The central vertical stress of the small coal pillar peaked at 14.30 MPa after the excavation of the adjacent face mining panel gateway. The vertical stress at the center of the small coal column peaked at 14.94 MPa at 10 m in front of the mining panel as the panel advanced. The vertical stresses peaked at 20.47 MPa and 22.11 MPa at 10 m and 20 m behind the mining panel, respectively. The peak value of the vertical stress in the small coal pillar increased and shifted to the side of the mining panel gob. The small coal pillar 10 m in front of the adjacent working face had a peak vertical stress of 24.49 MPa, 3.06 times the original rock stress when the adjacent mining panel was advanced. The uniaxial compressive strength of coal measured in the laboratory was 9.18 MPa, and the triaxial compressive strength was 22.43 MPa at a confining pressure of 4 MPa. After mining, the small coal pillar was destroyed, and the stability was difficult to maintain. It was therefore necessary to reduce the load and increase the strength to maintain the stability of the small coal pillar.

In the four stages of the excavation of the mining panel gateway, excavation of the adjacent mining panel gateway, retreating of the mining panel, and retreating of the adjacent mining panel, the distribution range of the central vertical stress nephogram of the small coal pillar changed from 12 ~ 14 MPa in green to 24 ~ 26 MPa in blue, and the superposition of the central vertical stress of the small coal pillar increased. When a small coal pillar is used to protect a roadway on two adjacent mining panels, the small coal pillar is affected by the superposition of four dynamical pressures from the excavation of the roadway and the advance of the mining panels on either side. Especially after mining, the pressure on small coal pillars increases dramatically. The superposition of pressure breaks up the small coal pillar and reduces the carrying capacity.

The vertical stress on the left side of the adjacent mining panel gateway peaked at 15.48 MPa after the gateway excavation. During the retreating of the mining panel, the vertical stress on the left side of the gateway peaked at 16.97 MPa at 10 m in front of the panel. The vertical stress peaked at 19.65 MPa and 20.84 MPa at 10 m and 20 m behind the mining panel, respectively, with a sharp increase of 34.63% in the vertical stress peak compared with the gateway excavation phase. When the adjacent panel was retracted, the peak value of the vertical stress increased to 24.68 MPa at 10 m in front of the panel, an increase of 59.43% compared with the gateway excavation phase.

The vertical stress nephogram of the left side of the adjacent mining panel gateway varied from 14 ~ 16 MPa in green to 24 ~ 26 MPa in blue, with a gradual increase in vertical stress during the three stages of the excavation of the adjacent mining panel gateway, retreating of the mining panel, and retreating of the adjacent mining panel. The front abutment pressure in the retreating phase of the adjacent panel had a large effect on the vertical stress on the left side, increasing the vertical stress distribution from 16 ~ 18 MPa to 24 ~ 26 MPa.

In summary, during the excavation of the roadway on both sides and the retreat of the two mining panels, the surrounding rock of the small coal pillar roadway was subjected to multiple dynamic pressures, the superposition of which increased the stress on the surrounding rock of the roadway. As one of the mining panels retreated, the roadway of the adjacent panel was severely affected by the mining, resulting in a dramatic increase in pressure on the adjacent roadway. Therefore, in order to reduce the superposition of stress in the roadway surrounding rock, cracks are prefabricated in the roof during the retreating of the mining panel to block the stress transfer so that the roof can collapse in time and reduce the mining influence on the pressure of the adjacent roadway, to reduce the superposition effect of stress in the small coal pillar roadway in different stages, and to make the retreating of the adjacent mining panel safe and efficient.

### Effect of energy accumulation on the surrounding rock of the small coal pillar roadway

The yield and failure of coal mass are accompanied by the accumulation, transfer, and release of elastic energy. When elastic energy is stored to a certain extent, it is released in a certain direction. Coal and rock mass deformation occurs when the released energy is greater than the critical value of the energy required for failure. When small coal pillars are used to protect a roadway, energy accumulation and conversion occur on the small coal pillars numerous times during the roadway excavation and mining panel retreat process. Thus, the effect of the accumulation of elastic energy on the small coal pillar was investigated, and the failure of the small coal pillar was analyzed in terms of energy.

The calculation formula of the elastic strain energy in the coal rock mass is

1 $${U^e} = \frac{1}{{2E}}\left[ {\sigma _1^2 + \sigma _2^2 + \sigma _3^2 - 2\mu ({\sigma _1}{\sigma _2} + {\sigma _2}{\sigma _3} + {\sigma _1}{\sigma _3})} \right]$$

where ${U^e}$ is the elastic strain energy density (kJ/m^3^); $E$ is the initial elastic modulus of the coal mass (GPa); $\mu$ is Poisson’s ratio of the coal mass; and ${\sigma}_{1}$, ${\sigma}_{2}$, and ${\sigma}_{3}$ are the principal stress (MPa).

The following formulas are used to calculate the shape-change energy density ${U^f}$and volume-change energy density ${U^v}$ in the coal mass :

2 $$\begin{gathered} {U_f} = \frac{{1 + \mu }}{{6E}}\left[ {{{({\sigma _1} - {\sigma _2})}^2} + {{({\sigma _2} - {\sigma _3})}^2} + {{({\sigma _3} - {\sigma _1})}^2}} \right] \hfill \\ {U^V} = \frac{{1 - 2\mu }}{{6E}}{({\sigma _1} + {\sigma _2} + {\sigma _3})^2} \hfill \\ \end{gathered}$$

The elastic strain energy of coal mass can be expressed by shape-change energy density and volume-change energy density, and Eq. (1) can be further expressed as:

3 $${U^e} = \frac{{1 - 2\mu }}{{6E}}{({\sigma _1} + {\sigma _2} + {\sigma _3})^2} + \frac{{1 + \mu }}{{6E}}\left[ {{{({\sigma _1} - {\sigma _2})}^2} + {{({\sigma _2} - {\sigma _3})}^2} + {{({\sigma _3} - {\sigma _1})}^2}} \right]$$

The principal stresses at each point in the coal mass can be obtained using a numerical simulation, and the elastic strain energy density, shape-change energy density, and volume-change energy density can be further calculated. The deformation and failure of small coal pillars can be judged by comparing the shape-change energy required to reach the strength limit and the volume-change energy required for the dynamical impact.

**Fig. 4:**
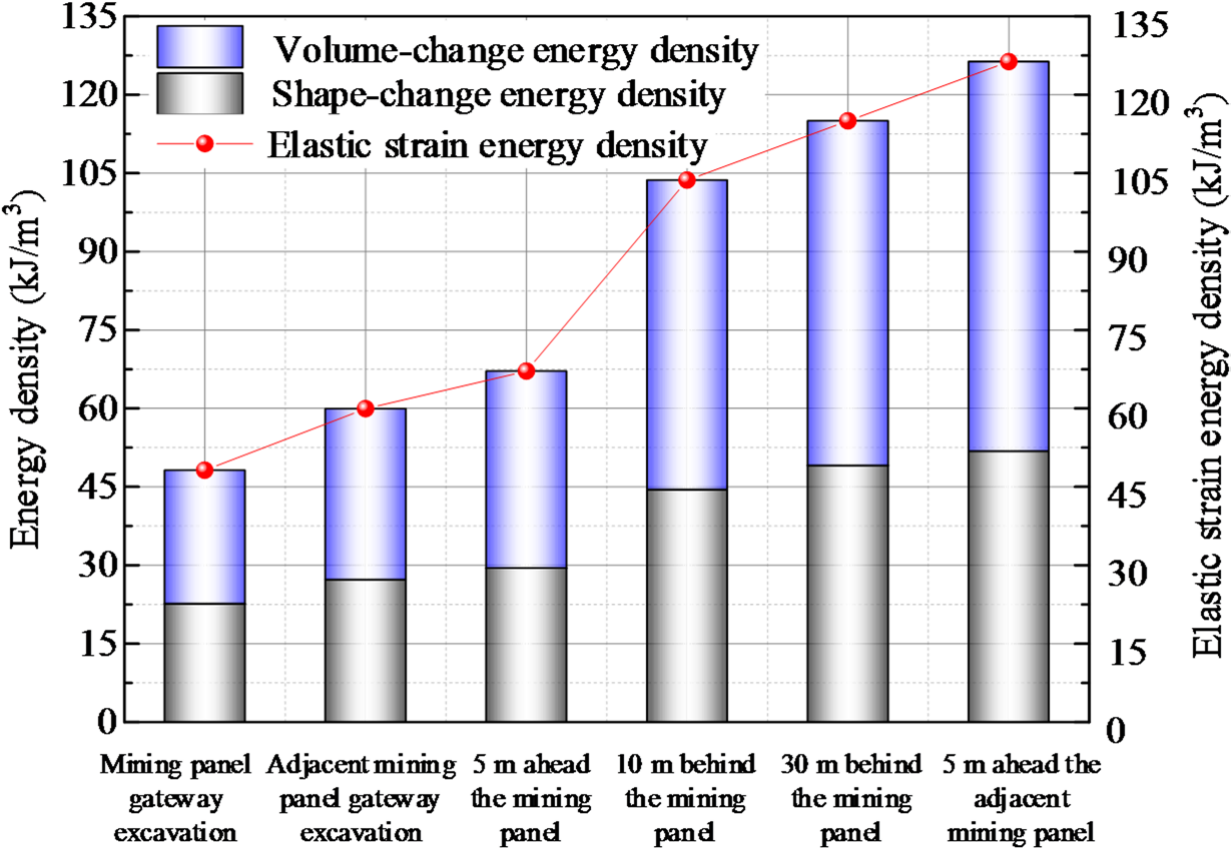
Energy accumulation process on the small coal pillar.

The energy at the central point of the small coal pillar was analyzed. Figure 4 illustrates the energy accumulation process on the small coal pillar during the excavation of the mining panel gateway, the excavation of the adjacent mining panel gateway, the retreat of the mining panel, and the retreat of the adjacent mining panel.

After the excavation of the adjacent mining panel gateway, the elastic strain energy density, shape-change energy density, and volume-change energy density were 48.16 kJ/m^3^, 22.64 kJ/m^3^, and 25.52 kJ/m^3^, respectively, at the central point of the small coal pillar. The elastic strain energy density at the central point of the small coal pillar increased to 59.93 kJ/m^3^, an increase of 24.44%. The shape-change energy density increased to 27.23 kJ/m^3^, an increase of 20.27%. The volume-change energy density increased to 32.70 kJ/m^3^, an increase of 28.13%. During the excavation on both sides of the small coal pillar, the energy on the small coal pillar increased under the influence of the driving dynamic pressure. Under uniaxial compression, the energy density of coal samples reaching the ultimate strength was 45.90 kJ/m^3^. The shape-change energy density at the center of the small coal pillar was smaller than the energy density of the coal samples reaching the ultimate strength, and no damage occurred to the small coal pillar.

When the mining panel was retreated, at the positions of the 5 m forward, 10 m backward, and 30 m backward panels, the elastic strain energy density increased by 39.35%, 115.23%, and 138.72% respectively, at the center of the small coal pillar; the shape-change energy density increased by 30.09%, 96.45%, and 116.79%, respectively; and the volume-change energy density increased by 47.57%, 131.88%, and 158.18%, respectively. As the mining panel retreated, the energy density on the small coal pillar increased, the energy accumulation increased significantly, and the energy density increase on the small coal pillar backward mining panel was large. After mining, the shape-change energy density at the central point of the small coal pillar was 49.08 kJ/m^3^, the volume-change energy density was 65.90 kJ/m^3^, and the small coal pillar center was destroyed. This indicates that the energy on the small coal pillar continued to accumulate under the load of the long overhanging roof at the side of the mining panel gob, causing constant deformation of the small coal pillar and difficulty in controlling its stability.

When the adjacent mining panel was retreated, at the position of the 5 m forward panel, the elastic strain energy density increased to 126.30 kJ/m^3^ at the central point of the small coal pillar; the shape-change energy density increased by 128.56% to 51.81 kJ/m^3^; and the volume-change energy density increased by 191.83% to 74.49 kJ/m^3^. With the retreat of the adjacent mining panel, the energy density on the small coal pillar increased under the front abutment pressure, the volume-change energy density increased by a large amount, and the energy on the small coal pillar accumulated further.

The minimum energy accumulation required for coal outburst is *E*_*min*_ *= ρv*^2^/2. Here, *ρ* is the average density of the coal, *ρ* = 1400 kg/m^3^, and *v* is the expulsion velocity of coal outburst. When *v*=10 m/s, the accumulated energy required for coal outburst is 70.00 kJ/m^3^. In the retreat of the adjacent mining panel, the volume-change energy density was greater than the minimum energy accumulation required for coal outburst at 5 m in front of the adjacent panel. Due to the accumulation of high energy, there was a tendency for rock burst in the area affected by the front abutment pressure when the adjacent panel was mined.

In summary, energy continued to accumulate on the small coal pillar as it was subjected to multiple dynamic pressures during roadway excavation and mining panel retreat on either side of it. After the mining panel retreated, the high accumulated energy caused the failure of the small coal pillar and continuous deformation of the adjacent gateway. As the adjacent mining panel retreated, energy continued to accumulate on the small coal pillars in areas affected by forward abutment pressure, leading to a tendency for rock rupture, which severely affected the safe mining of the panel. Consequently, cracks formed in the roadway roof near the pillar to reduce the accumulation of energy on the small coal pillar.

### Deformation law of the adjacent mining panel gateway during mining panel retreat

**Fig. 5:**
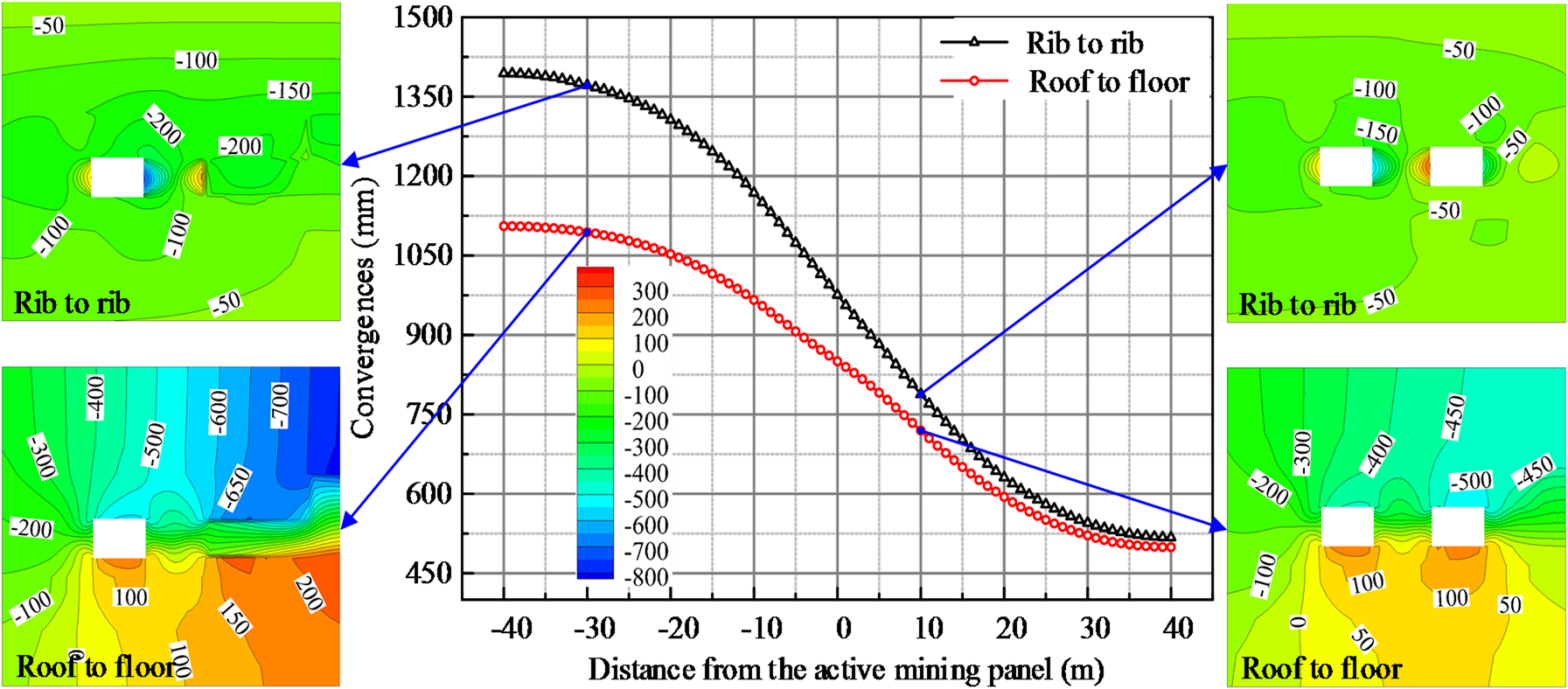
A adjacent mining panel gateway deformation curves and displacement nephogram.

The adjacent mining panel gateway deformation curves and displacement nephogram recorded during the mining panel retreat are shown in Fig. 5. From the 40 m forward mining panel to the 40 m backward mining panel, the deformation of the adjacent mining panel gateway increased. During the entire deformation phase, the roof-to-floor deformation on the adjacent mining panel gateway increased by 121.39%, from 499.3 mm to 1,105.4 mm; the rib-to-rib deformation on the adjacent mining panel gateway increased from 517.5 mm to 1394.1 mm, an increase of 169.39%. In the 40 m backward mining panel, the deformation increase was considerably larger in the adjacent mining panel gateway. It is typical to increase the number of individual hydraulic props in order to control the deformation of the adjacent mining panel gateway. The density of the individual hydraulic props near the side of the small coal pillar is large, and the spacing of the hydraulic props is small, thus increasing the strength of the support. However, the limited support capacity of a single hydraulic prop makes it difficult to control the roadway deformation. At the same time, the increase in the number of individual hydraulic props makes it difficult for personnel and equipment to enter and exit, which critically impacts efficient mining.

From the 10 m forward mining panel to the 20 m backward mining panel, the roof nephogram of the adjacent mining panel gateway increased from green in the 400–450 mm range to light blue in the 650–700 mm range; the floor nephogram essentially did not change. The nephogram of the small coal pillar side increased from light blue in the 500–550 mm range to dark blue in the 750–800 mm range; the solid coal wall side nephogram fundamentally did not shift. The increase in the deformation of the adjacent mining panel gateway was mainly caused by the increase in the deformation of the roof and the small coal pillar side, while the increase in the deformation of the floor and the solid coal side was small. The proximity of the two sides of the gateway was greater than that of the roof and floor. The deformation on the small coal pillar side was certainly greater than that on the solid coal side. The subsidence of the roof near the mining panel gob side was greater than the subsidence on the mining panel side.

To sum up, after the mining panel retreated, the lateral hanging roof was large, resulting in a high additional load on the adjacent mining panel gateway and a weak bearing capacity of the small coal pillar. This led to a sharp increase in the deformation of the adjacent gateway, making it difficult to ensure the normal retreat of the adjacent mining panel. Pre-splitting blasting is used to adjust the roof structure, reduce the load on the adjacent gateway, and optimize the stressful environment.

## Migration and fracture laws of overlying rock with prefabricated cracks in the roof of a small coal pillar roadway

The impact of prefabricated cracks in the roof of a small coal pillar roadway on the overburden migration law and the fracture structure was investigated using physical simulation methods, thus providing a basis for the roof control of small coal pillar roadways under stress superposition.

### Physical simulation test parameter design and test scheme

**Fig. 6:**
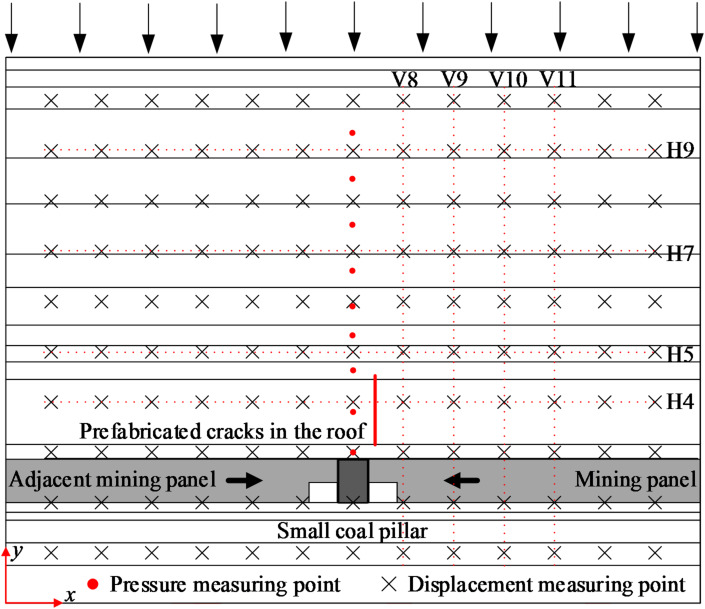
Physical simulation test scheme and measuring point arrangement.

A physical simulation was used to investigate the caving morphology of the overlying rock and the stress distribution properties of the surrounding rock after a crack was prefabricated in the roadway roof. The size of the physical simulation frame was length × width × height = 1380 mm×120 mm×1100 mm. The simulation scheme and the arrangement of the measurement points are shown in Fig. 6. The pressure measurement points were arranged in the roof area of the small coal pillar. A total of nine pressure measurement points were arranged, with the number of measurement points ranging from P1 to P9 from bottom to top. Horizontal and vertical survey lines were aligned every 100 mm on the model. The horizontal survey lines were numbered H1 to H10 from bottom to top, and the vertical survey lines were numbered V1 to V13 from left to right. The intersection of the horizontal and vertical geodesics was the displacement point.

In the physical simulation, the geometry ratio between the model and the prototype was *C*_*l*_=1/80; the width and height of the roadway in the model were 56.3 mm and 40.0 mm, respectively; and the width of the small coal pillar was 62.5 mm. The average density of the prototype rock was 2500 kg/m^3^, the average density of the model-similar material was 1500 kg/m^3^, the bulk density ratio was *C*_*r*_ = 3/5, and the stress similarity ratio was *C*_*σ*_ *= C*_*l*_*×C*_*r*_ = 1/133. For the unmodeled overburden strata in the physical simulation, the actual in-situ overburden pressure is 6.4 MPa. Accordingly, an additional stress of 0.048 MPa is applied to the model. River sand was chosen as the aggregate, light calcium carbonate and gypsum as the cement, mica powder as the layered material, and water as an auxiliary. The physical model was laid out layer by layer according to the design ratio. Simulated stratification and intensity matching for similar materials used for testing are shown in Table 2. Lubricating oil was uniformly applied to the inner walls of the frames on both sides of the model, followed by the placement of plastic films. This procedure serves to minimize lateral frictional resistance during strata movement, thereby enabling more accurate simulation of overburden migration under field conditions.

**Table 2:** Simulated delamination and strength ratio of similar materials.

Lithology name	Prototype (m)		Model (cm)		Layer number	Compressive strength (MPa)		Proportion
	Layer thickness	Gross thickness	Layer thickness	Gross thickness		Prototype	Model	Sand/lime/gypsum
Siltstone	2.00	86.85	2.50	108.56	1	45.80	0.34	637
Fine sandstone	2.70	84.85	3.38	106.06	1	61.04	0.46	537
Mudstone	3.50	82.15	4.38	102.69	1	32.40	0.24	655
Sandy mudstone	7.80	78.65	9.75	98.31	3	37.50	0.28	646
Medium sandstone	7.30	70.85	9.13	88.56	3	58.62	0.44	546
Sandy mudstone	8.00	63.55	10.00	79.44	3	37.50	0.28	646
Mudstone	5.30	55.55	6.63	69.44	2	32.40	0.24	655
Sandy mudstone	6.00	50.25	7.50	62.81	2	37.50	0.28	646
Mudstone	3.25	44.25	4.06	55.31	1	32.40	0.24	655
Fine sandstone	2.60	41.00	3.25	51.25	1	61.04	0.46	537
Mudstone	2.80	38.40	3.50	48.00	1	32.40	0.24	655
Fine sandstone	10.35	35.60	12.94	44.50	4	98.31	0.82	528
Sandy mudstone	2.35	25.25	2.94	31.56	1	37.01	0.28	646
Coal	6.90	22.90	8.63	28.63	1	9.18	0.08	773
Mudstone	1.55	16.00	1.94	20.00	1	32.40	0.24	655
Fine sandstone	1.25	14.45	1.56	18.06	1	61.04	0.46	537
Mudstone	3.60	13.20	4.50	16.50	1	32.40	0.24	655
Sandy mudstone	3.60	9.60	4.50	12.00	1	37.50	0.28	646
Fine sandstone	6.00	6.00	7.50	7.50	1	61.04	0.46	537

**Fig. 7:**
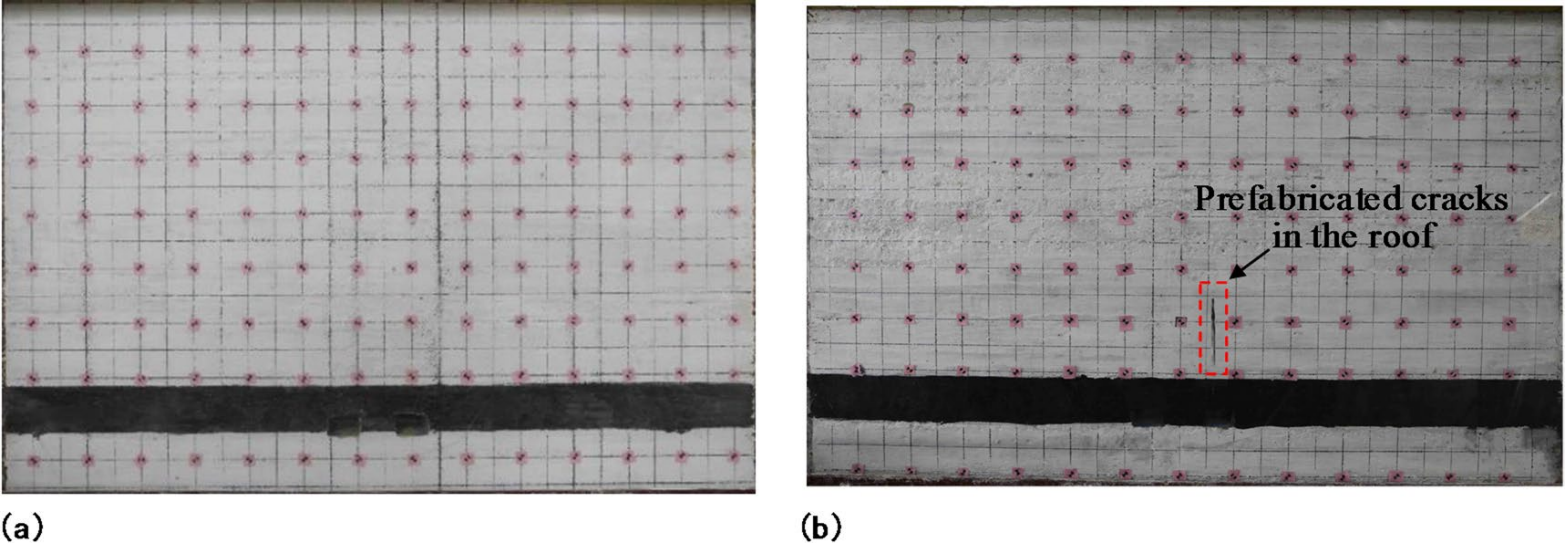
The model laying completed pictures: **(a)** no prefabricated cracks in the model’s roof; **(b)** prefabricated cracks in the model’s roof.

In total, two similar models were laid, and the completed similar model is shown in Fig. 7. In Fig. 7 (a), there is no prefabricated cracks in the model’s mining panel gateway roof. In Fig. 7 (b), there are prefabricated cracks in the model’s gateway roof. After the gateway excavations on either side of the small coal pillar were completed, cracks were prefabricated in the roof of the mining panel gateway. The prefabricated cracks are designed to induce early fracture of the main roof through pre-splitting blasting. The distance from the crack to the small coal pillar is determined based on the installation and operational space required for the field drilling equipment. Adequate clearance must be maintained to ensure proper setup and operation of the drilling rig. Under these engineering and construction constraints, the distance between the crack and the small coal pillar was set to 12.5 mm, and the height of the crack was set to 103.5 mm.

During testing, the mining panel first retreated from right to left, and the adjacent mining panel retreated from left to right after the mining panel retreat was completed, with each mining step being 5 cm. A statical strain indicator was used to continuously collect pressure capsule data. At the same time, a high-definition camera was used to photograph the collapse of the model overburden every 8 s. The camera was fixed at the same position and was prevented from moving during the taking of the photograph, thus ensuring an accurate calculation of the overburdened migration.

### Influence of the prefabricated cracks in the roof on the overburdened vertical fracture structure

**Fig. 8:**
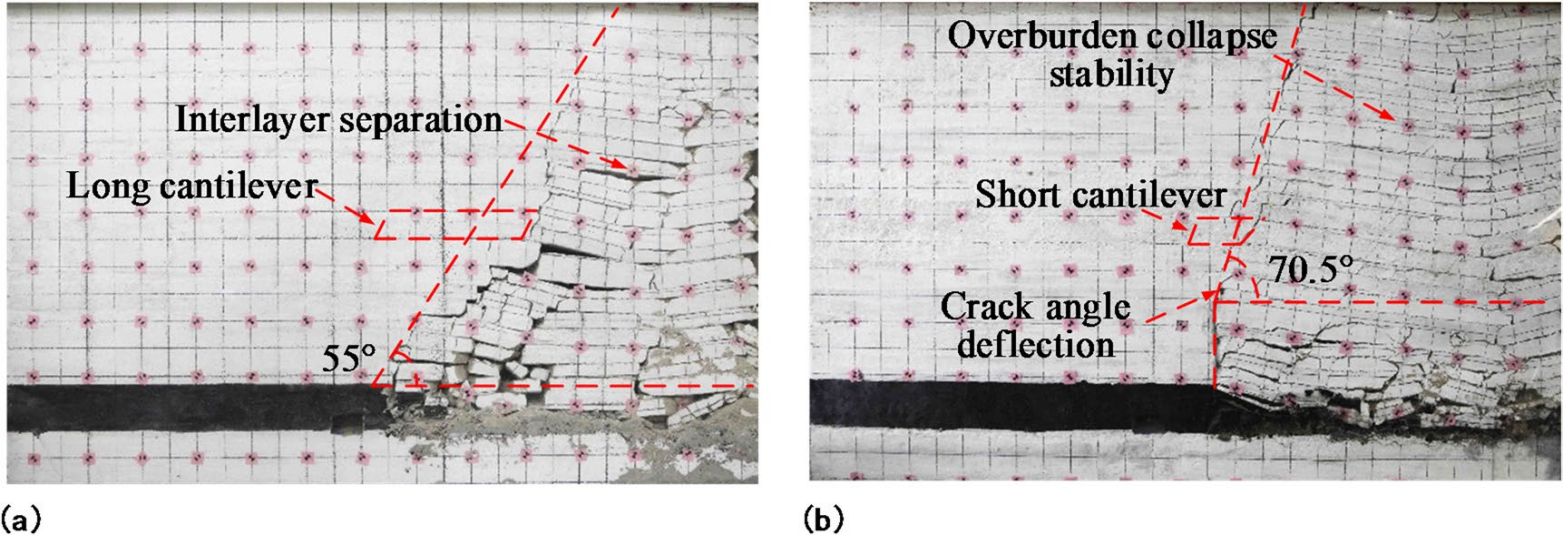
Overburden caving structure after mining panel retreat: **(a)** no prefabricated cracks in the model’s roof; **(b)** prefabricated cracks in the model’s roof.

**Fig. 9:**
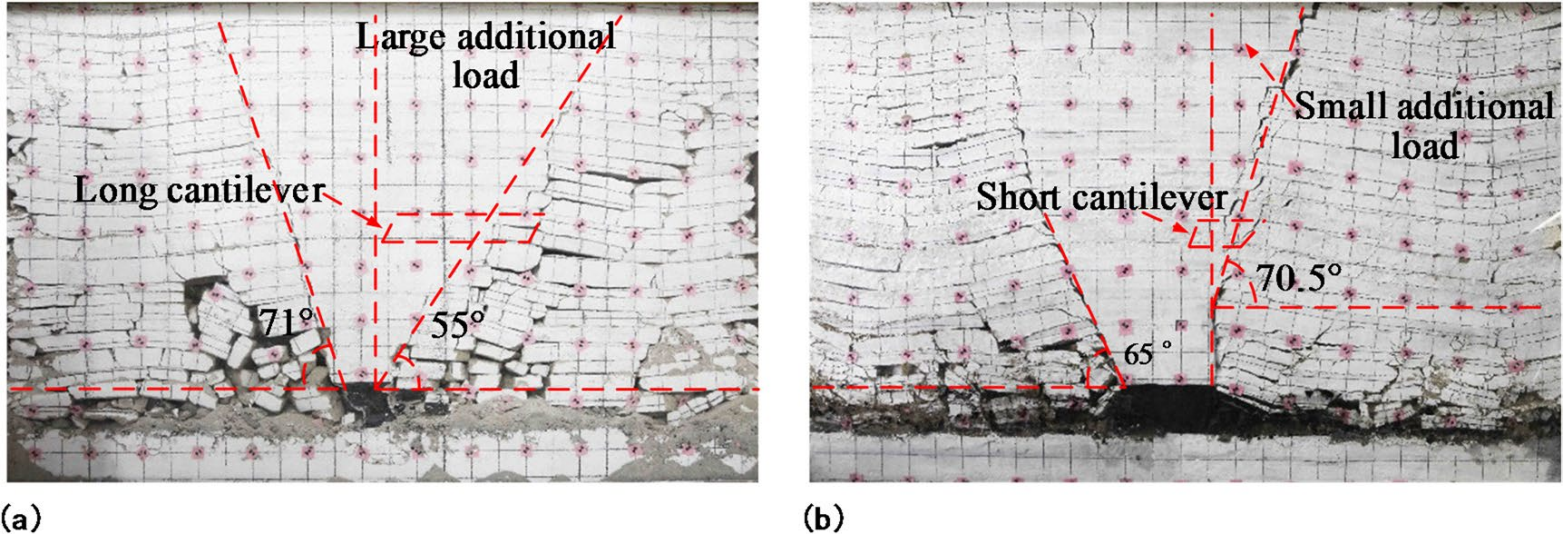
Overburden caving structure after adjacent mining panel retreat: (**a**) no prefabricated cracks in the model’s roof; (**b**) prefabricated cracks in the model’s roof.

With the retreat of the mining panels, overburdened caverns in the gob gradually developed into the upper strata, showing regular fractures and the formation of articulated structures between the fractured blocks. The overburdened fracture structures of the mining panel and adjacent mining panel are shown in Figs. 8 and 9, respectively.

When the model’s roof was not prefabricated, there were cracks and insufficient overburden caving, resulting in uneven subsidence between the strata and inter-layer separation. Additionally, there were lateral cracks in the caving overburden, a minor compaction degree, and obvious gaps between broken rock in the gob, which could not effectively support the overburden. The roof strata caving angle was about 55° due to the great basic roof strength, excellent roof integrity, and difficulty of collapse for the rock strata. In the overlying bond layer, a long cantilever structure with a length of approximately 25 cm was formed; this allowed small coal pillars to bear a large load but exerted pressure on the surrounding rock detrimental to roadway maintenance and mining panel retreat.

When the model roof was prefabricated with cracks, there was no inter-layer separation, and the overlying rock cavity remained stable. At the height of the prefabricated crack in the roof, where the formation collapsed along the crack, the density of the caving rock filling was favorable, supporting the overlying caving rock and reducing the load on the adjacent mining panel gateway. Above the range of cracks in the roof strata, a diagonal upward extension of the caving line occurred along with a caving angle of about 70.5° for the roof strata. The overhang length of the overlying bond strata was significantly shortened, forming a short cantilever structure with a length of about 13 cm, representing a 48% reduction in overhang length after the formation of prefabricated fractures. This suggests that roof prefabricated cracks increase the collapse of rock formations, allowing the cavernous rock to fill the gob and support the remaining roof. Additionally, an increased angle at which the roof caves in reduces the length of the overhanging roof and decreases the stresses on and deformations of the roadway in the adjacent mining panel, facilitating the maintenance of the roadway.

After the retreat of the adjacent mining panel, the roof caving angle with no prefabricated cracks and with prefabricated cracks were 71° and 65°, respectively. The roof caving angle after prefabricating cracks were minimal. When the model roof was prefabricated with cracks, the roof overhang of the mining panel was short, and the load on the roof of the adjacent mining panel was minor. The strength and integrity of the adjacent mining panel roof were better with prefabricated cracks than without them. Consequently, the roof rock had a smaller caving angle.

After analyzing the above, it can be observed that prefabricated cracks alter the rock caving angle and the structure of the hanging roof. Following the creation of cracks in the roof, it collapsed at a steep angle, resulting in a short overhang of the overburden and minimal load on the adjacent gateway.

### Influence of the prefabricated cracks in the roof on overburden deformation

**Fig. 10:**
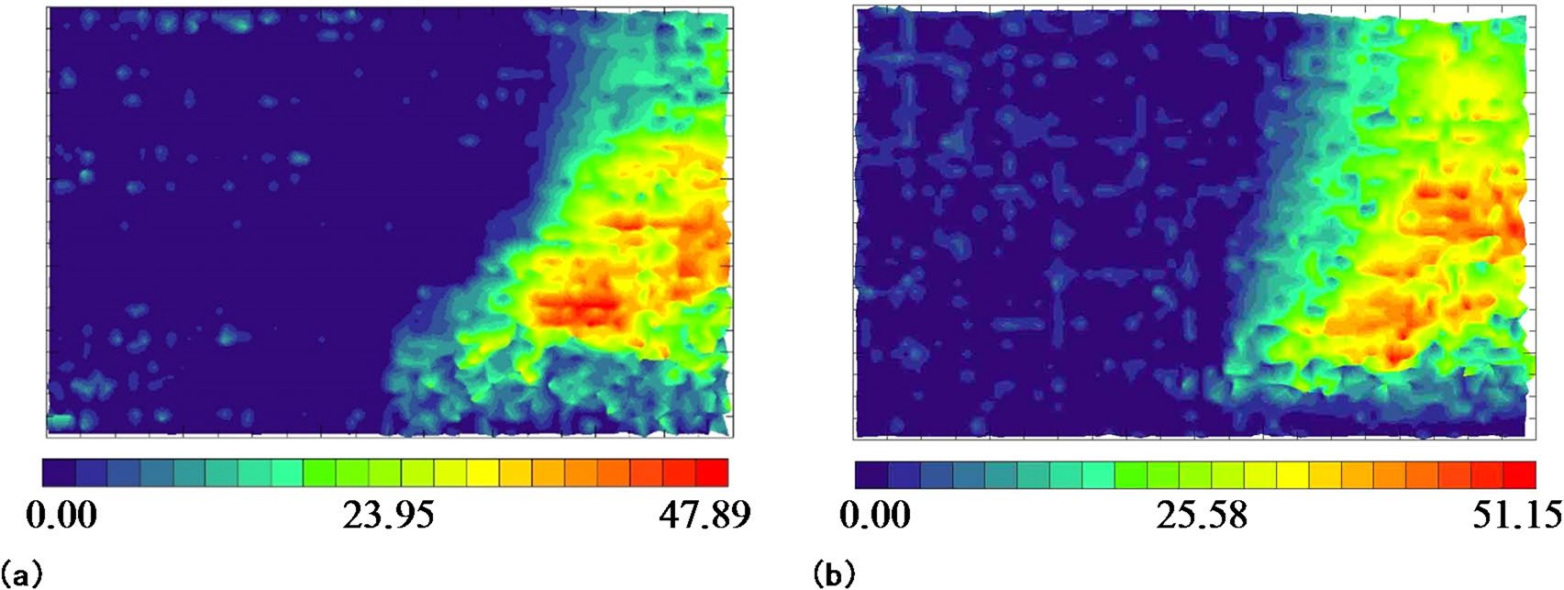
Overburden rock displacement nephogram after mining panel retreat:**(a)** no prefabricated cracks in the model’s roof; **(b)** prefabricated cracks in the model’s roof.

Photogrammetry was used to study the deformation laws of the overlying rock after the mining panel retreated on either side of the small coal pillar. Figure 10 shows the overburdened rock displacement nephogram after the mining panel retreat. The displacement was fundamentally the same in the lower strata, ranging from 40 mm to 50 mm. The prefabricated cracks in the model roof had a slight effect on the subsidence of the lower strata. For the higher strata, the displacement ranged from 10 mm to 20 mm when there were no prefabricated cracks in the model roof. After prefabricating the cracks in the model roof, the displacement of the upper strata ranged from 25 mm to 35 mm. The displacement of the upper strata increased, and, within this range, it was observed that there was a significant increase in displacement. It can be seen that prefabricated cracks in the roof have a slight effect on the displacement of the lower strata while causing more substantial subsidence in the higher strata under their influence, in terms of both displacement amount and area. Consequently, prefabricated cracks in the roof lead to a greater compaction of gob and shorten its stability time.

**Fig. 11:**
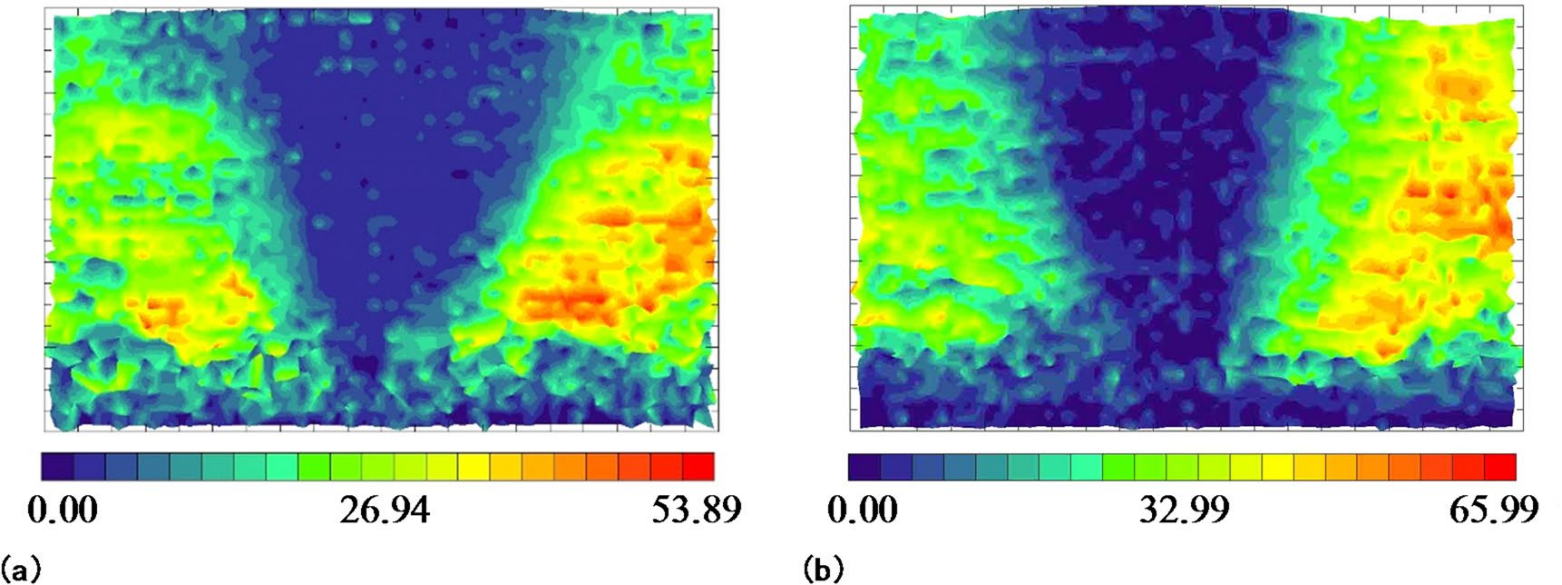
Overburden rock displacement nephogram after adjacent mining panel retreat: **(a)** no prefabricated cracks in the model’s roof; **(b)** prefabricated cracks in the model’s roof.

Figure 11 depicts the displacement nephogram of overburdened rock after the adjacent mining panel retreated. The caving rock displacement of the mining panel gob continued to increase under the influence of dynamic pressure as the adjacent mining panel retreated, while there was only a minor increase in the case of prefabricated cracks in the roof. This indicates that the mining panel gob was fundamentally stable when the adjacent mining panel retreated. The overall displacements of the adjacent mining panel gob caving rock were not extremely different, ranging from 40 to 50 mm in the lower layers and from 20 to 30 mm in the upper layers. The prefabricated cracks in the model roof had a slight effect on the deformation of the adjacent mining panel gob caving rock.

The prefabricated cracks in the model roof had a significant influence on the deformation of the upper strata of the mining panel gob, leading to an increase in deformation and easier collapse. This shortened the stability time of the gob caving rock, which was beneficial for maintaining the roadway of the adjacent mining panel. The prefabricated cracks had a minor effect on the deformation of the lower strata in the mining panel gob and on the overlying strata in the adjacent mining panels. The displacement nephogram of overburdened rock remained fundamentally unchanged.

**Fig. 12:**
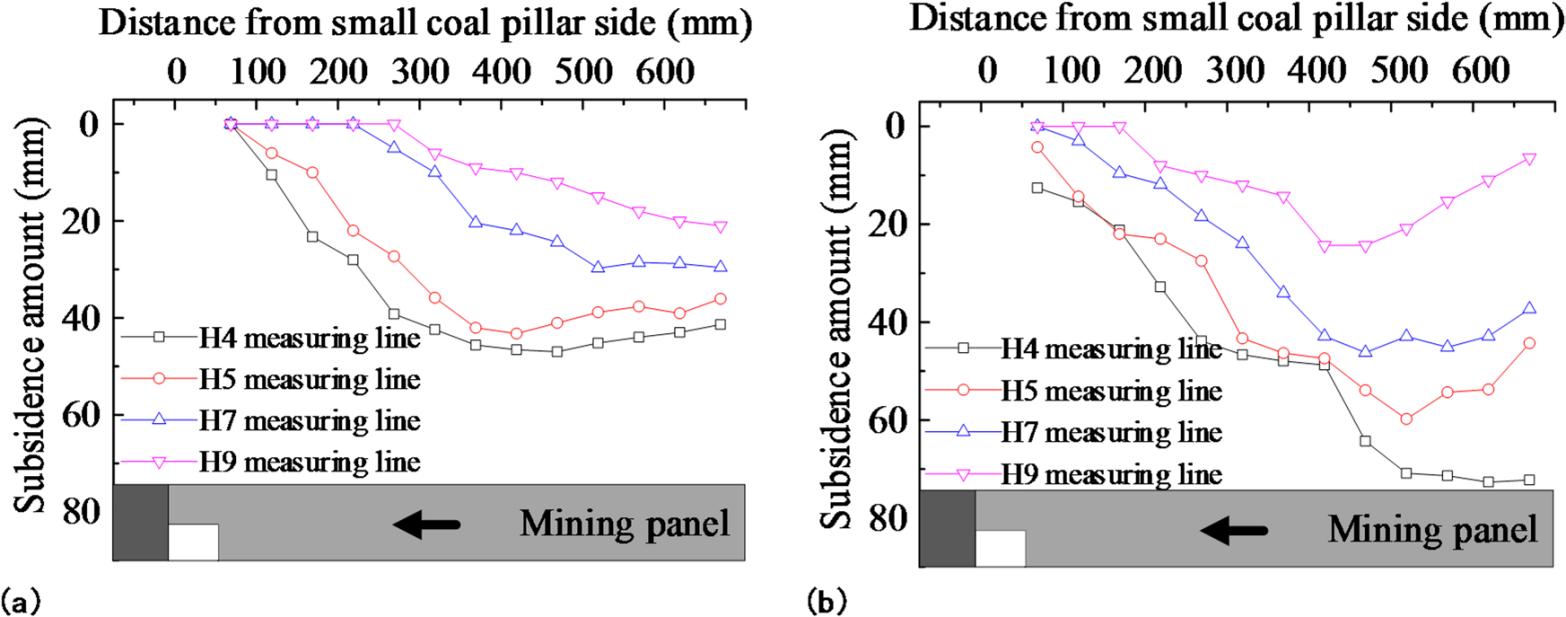
Survey line displacement variation after mining panel retreat: **(a)** no prefabricated cracks in the model’s roof; **(b)** prefabricated cracks in the model’s roof.

**Fig. 13:**
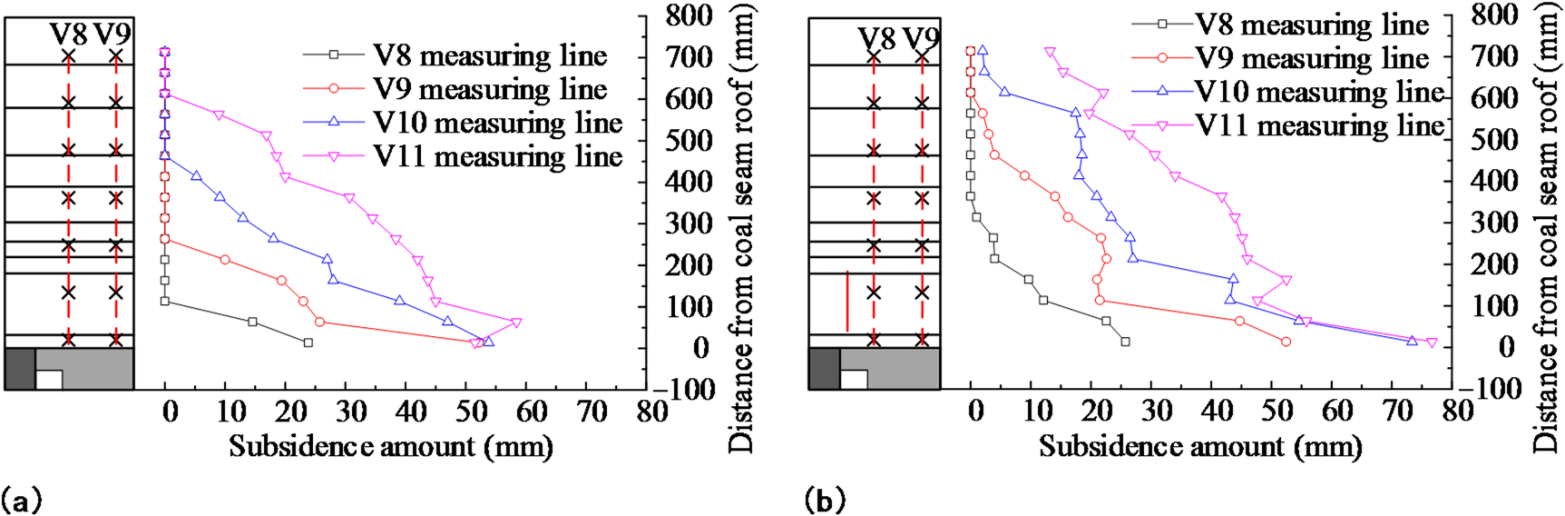
Vertical line displacement variation after mining panel retreat: **(a)** no prefabricated cracks in the model’s roof; **(b)** prefabricated cracks in the model’s roof.

The effect of prefabricated cracks on the displacement of the overburden after the retreat of the mining panel was investigated by analyzing the horizontal and vertical displacement lines. The selected horizontal lines were H4, H5, H7, and H9, with distances of 113.7 mm, 213.7 mm, 413.7 mm, and 613.7 mm from the line to the coal seam, respectively. The vertical survey lines V8, V9, V10, and V11 were chosen, with vertical distances of 56.3 mm, 156.3 mm, 256.3 mm, and 356.3 mm from the survey line to the small coal pillar, respectively. Figures 12 and 13 display the variation curves of the horizontal and vertical line displacements following the retreat of the mining panel.

In Fig. 12, it can be observed that the maximum displacements of the measured lines H4, H5, H7, and H9 were 46.98 mm, 43.24 mm, 32.76 mm, and 21.35 mm, respectively, when there were no prefabricated cracks in the model roof. The maximum displacements of each measured line were 72.70 mm, 59.78 mm, 41.22 mm, and 22.32 mm when pre-fabricated cracks were present. The maximum displacement increased by 54.75%, 35.25%, 25.82%, and 4.54% compared with the case without prefabricated cracks. The measured H4 line showed the largest increase under the effects of the prefabricated cracks, while the H9 line showed the smallest displacement increase. The higher the surveyed line, the smaller the increase in displacement affected by the prefabricated cracks. In the strata above survey line H9, displacement was nearly unaffected by the prefabricated cracks. Therefore, the distance between the highest strata affected by prefabricated cracks and the coal seam was 613.7 mm, corresponding to a site height of 49.10 m, which was approximately 5.93 times the height of the prefabricated cracks.

Figure 13 shows that the roof displacement decreased as the distance from the coal seam roof increased. When the roof was not prefabricated with cracks, there were no sinking points on the survey lines of V8, V9, V10, and V11. Subsidence occurred at all survey points after the roof was prefabricated with cracks. The greater the vertical distance between the survey line and the side of the small coal pillar, the less the displacement was affected by the prefabricated cracks. When the vertical distance between the survey line and the small coal pillar was greater than 356.3 mm, a minor effect of the cracks was observed. Therefore, the horizontal influence of the prefabricated cracks was about 356.3 mm, corresponding to a horizontal distance of 28.50 m on site.

According to the analysis above, the higher the stratum, the greater the horizontal distance from the prefabricated crack and the less the displacement is affected by it. The prefabricated crack had a vertical influence range of about 49.10 m, which was 5.93 times its height, and a lateral influence range of about 28.5 m.

**Fig. 14:**
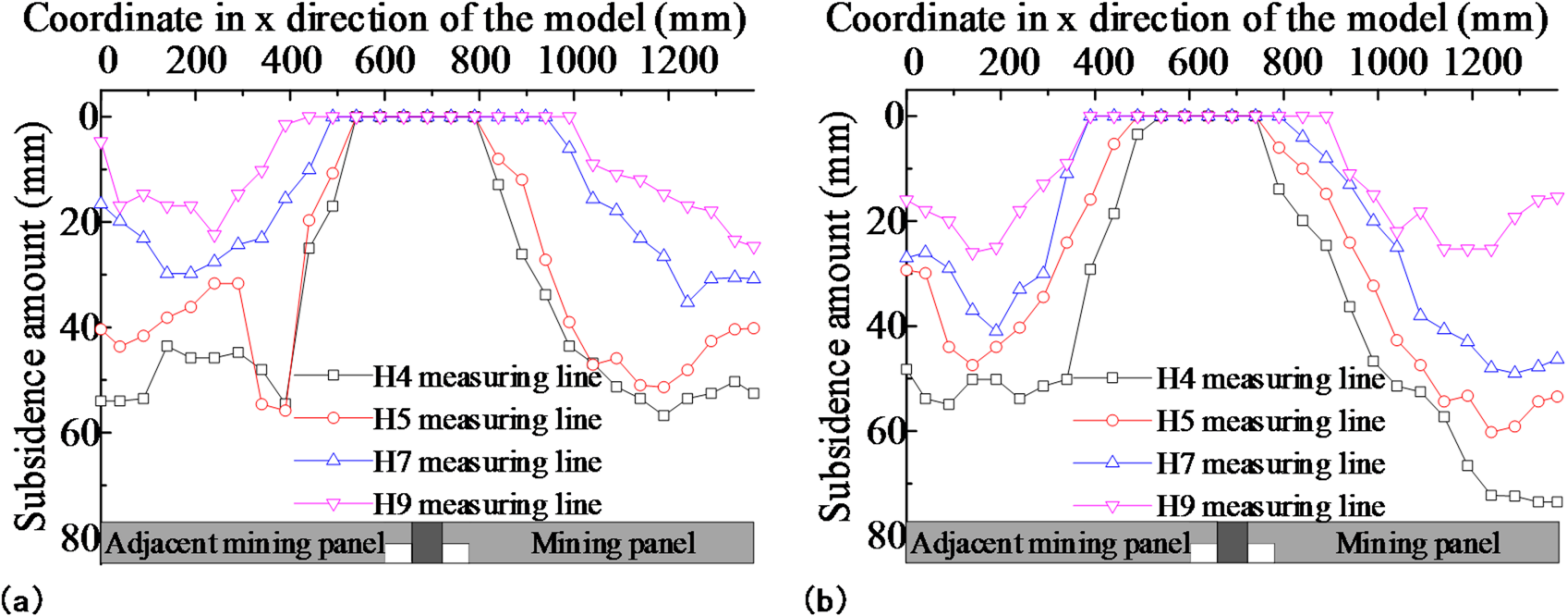
Survey line displacement variation after adjacent mining panel retreat:**(a)** no prefabricated cracks in the model’s roof; **(b)** prefabricated cracks in the model’s roof.

Figure 14 shows the displacement variation curves of the horizontal measurement lines H4, H5, H7, and H9 after the retreat of the adjacent mining panel. The displacement of the mining panel gob continued to increase as the adjacent mining panel retreated. When there were no prefabricated cracks in the model roof, the maximum displacement of each measurement line increased by 20.84%, 18.78%, 7.66%, and 6.09%, respectively. The maximum displacement of each measurement line increased by 1.10%, 5.74%, 4.29%, and 4.48% when there were prefabricated cracks in the model roof. The displacement of the measurement line slightly increased after prefabricating a crack. This indicated that, after prefabricating a crack in the roof, the caving rock at the mining panel gob fundamentally collapsed and stabilized as the adjacent mining panel retreated.

However, when the roof does not have prefabricated cracks, the caving rock at the mining panel gob will significantly sink under the influence of the mining dynamic pressure of the adjacent mining panel. The subsidence disturbance of the overlying rock will affect the gateway of the adjacent mining panel and further increase its deformation, affecting efficient retreat. The trends in the variability and displacement of each measurement line in the adjacent mining panel gob were fundamentally similar. Without prefabricated cracks in the roof, the maximum displacements of each measurement line were 53.51 mm, 43.64 mm, 29.78 mm, and 22.42 mm. With prefabricated cracks in the roof, the maximum displacements of each measured line were 54.91 mm, 47.45 mm, 40.47 mm, and 26.13 mm. The prefabricated crack had a minor effect on overlying rock displacement in the adjacent mining panel gob.

After prefabricating cracks in the roof of the mining panel gateway roof, the overlying rock cavern remained stable. The retreat of the adjacent mining panel had a minor effect on rock-caving disturbances in the mining panel gob, which favored the retreat of the adjacent mining panel.

### Stress distribution of small coal pillar roof after prefabricating cracks

**Fig. 15:**
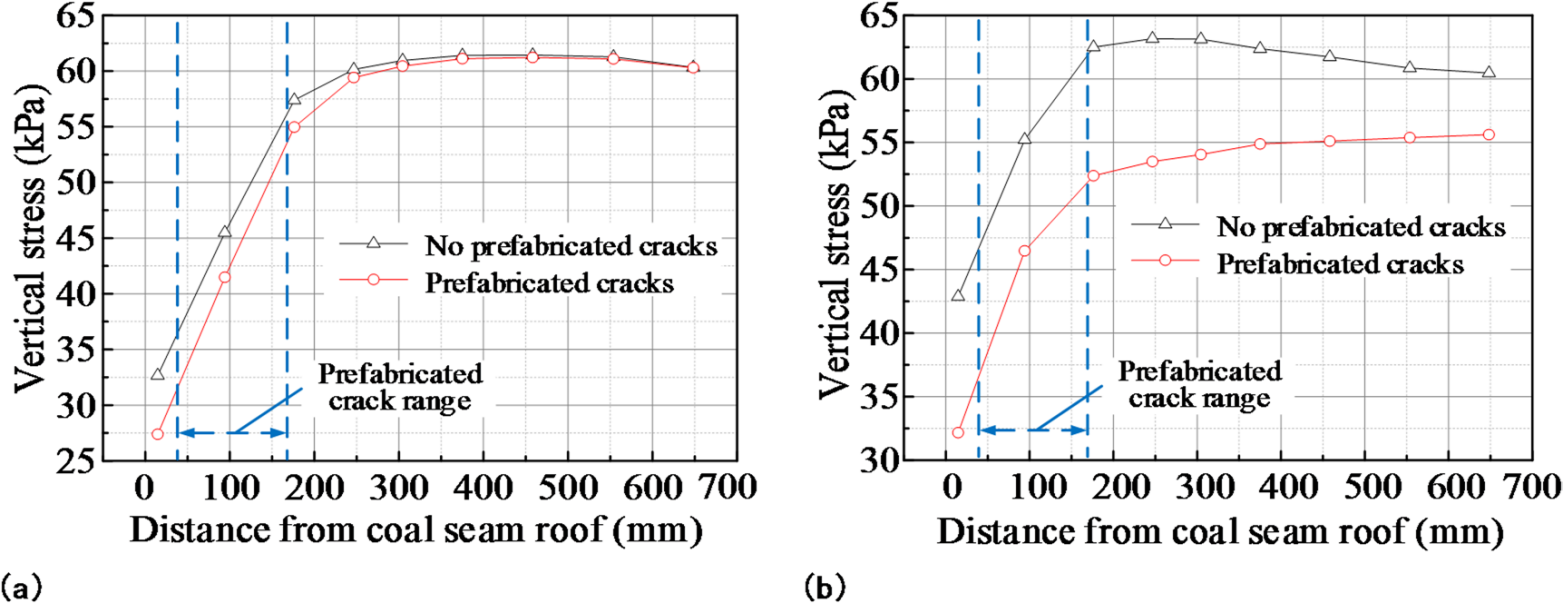
Variation curve of roof vertical stress in small coal pillar area: **(a)** no retreat of the mining panel; **(b)** retreat of the mining panel.

The pressure on the surrounding rock in the roadway was redistributed after prefabricating cracks in the mining panel gateway roof. In order to study the influence of prefabricated cracks on roof stress in small coal pillar areas, pressure boxes were arranged at different levels of the roof strata to analyze the change in the vertical stress in different strata. This revealed the distribution law of stress in small coal pillar roof areas after prefabricating cracks. A variation curve of the vertical stress of the roof in the small coal pillar region is shown in Fig. 15.

The vertical stress in the roof area of the small coal pillar increased with the increase in the roof height and eventually became stable, as shown in Fig. 15 (a). After prefabricating cracks in the main roof, the vertical stress decreased due to their effect, with the cracks affecting vertical stress within 2.9 times their height. The average reduction in vertical stress over the crack height range was 12.48%, while outside this range, it decreased by an average of 1.06%. Within the crack height range, there was a considerable decrease in vertical stress. The cracks affected the distribution of vertical stress within a certain height range, beyond which it remained unaffected by the prefabricated cracks.

It can be seen in Fig. 15 (b) that, after the mining panel retreated, the vertical stress in the roof area of the small coal pillar increased with an increase in the roof height and finally tended to be stable. When the roof was not prefabricated with cracks, the vertical stress on the roof within the area of the small coal pillar increased after mining. This was because, after mining, the length of the roof overhang increased, leading to an increase in load and, subsequently, an increase in the vertical stress on the roof. Within 250 mm of the roof, the vertical stress showed an average increase of 16.64%. Above 250 mm, the vertical stress showed an average increase of 1.36%. The vertical stress substantially increased within a range of 250 mm from where it reached up to a distance of 20 m field range. The effect of load on the roof within the small coal pillar area decreased with increasing height. When the roof had prefabricated cracks, the vertical stress on the roof increased by 14.72% in the 0–150 mm range after mining. Above 150 mm, the vertical stress on the roof decreased by an average of 8.93%. As the height of the roof increased, there was little change in the degree of vertical stress reduction. This was because the load on the roof suspension did not change much as the height of the roof increased. After mining, the prefabricated cracks in the roof reduced the vertical stress in the small coal pillar area by an average of 14.21% compared with the roof without the prefabricated cracks. The prefabricated cracks in the roof reduced the load on the overhanging roof and increased the ability of the fractured rock mass in the gob to support it, thereby reducing the vertical stress on the roof in the small coal pillar area. In summary, the pre-fabricated cracks reduced the vertical stress on the small coal pillar area’s roof, facilitating the maintenance of the small coal pillar and the adjacent mining panel gateway.

## Field application

**Fig. 16:**
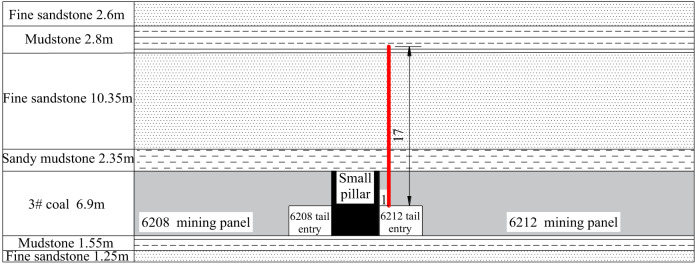
Schematic diagram of directional pre-splitting with a deep hole in the roof of the 6212 tail entry.

An advanced deep-hole directional pre-splitting technology scheme was designed for the 6212 tail entry of the Wangzhuang Coal Mine, based on the engineering geology of the 6212 mining panel and 6208 mining panel, the width of the small coal pillar is 5 m. Figure 16 shows a schematic of the technical scheme. Deep-hole directional pre-splitting pressure relief technology was implemented in the roof of the 6212 tail entry to create prefabricated cracks in the roof. The detailed directional pre-splitting parameters were as follows: the vertical drilling depth is 17 m, the drilling diameter is 55 mm, the diameter of the emulsion explosive is 35 mm, the charge length is 10 m, the total length of the directional shaped charge tube is 10 m, and the charge weight is 10 kg. With the retreat of the 6212 mining panel, the connection between the roof of the 6208 tail entry and the 6212 mining panel had been severed. After retreating the 6212 mining panel, the overhanging length of the main roof was reduced, optimizing the pressure environment of the surrounding rock for the protection of the small coal pillar and the 6208 tail entry.

**Fig. 17:**
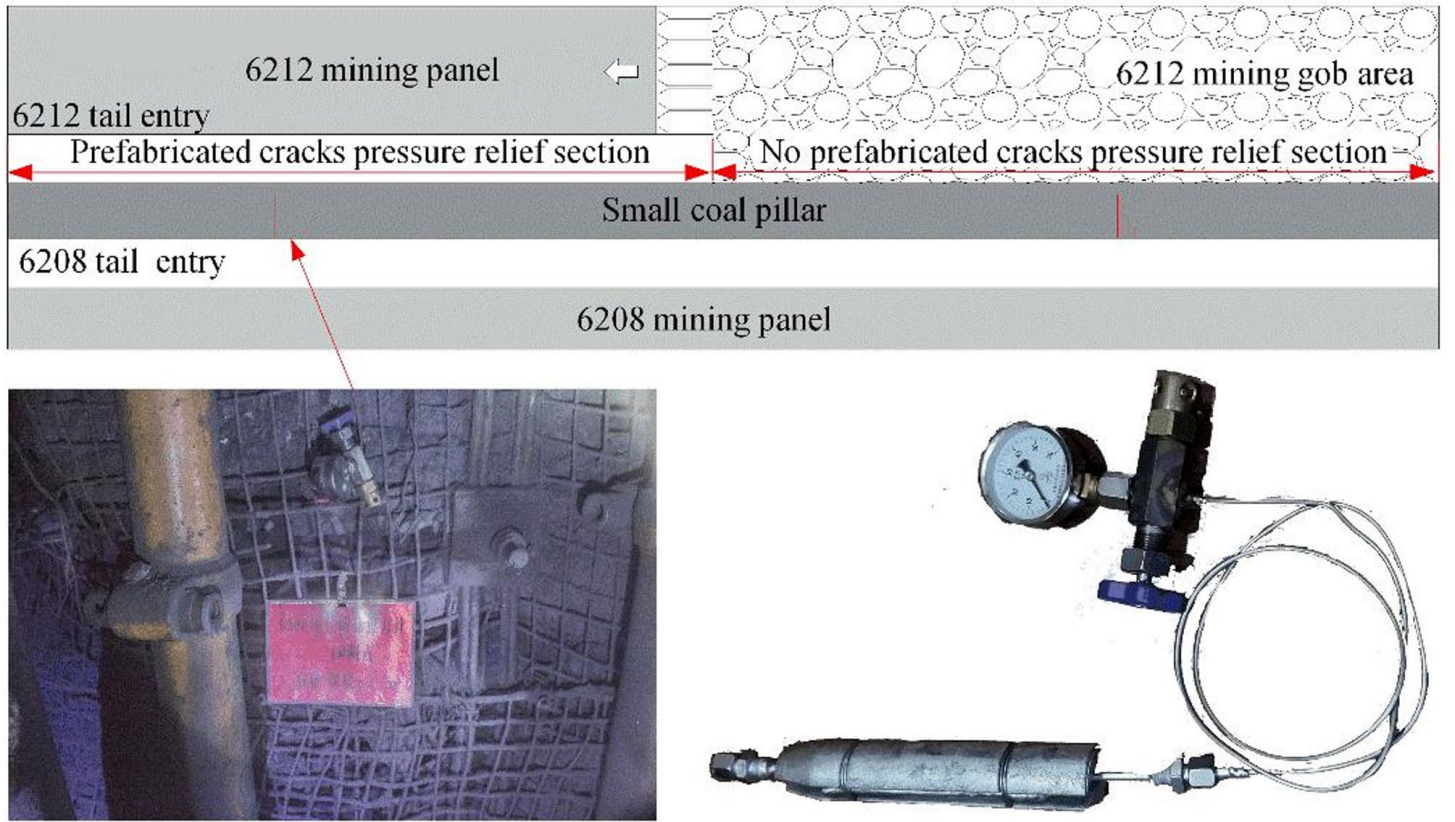
Schematic diagram of the stress monitoring scheme for small coal pillar.

A set of borehole stress measurement stations was arranged in the no prefabricated crack relief section and the prefabricated crack relief section. The stress variation law of the small coal pillar was monitored during the retreat of the 6212 mining panel. Figure 17 shows a schematic of the stress monitoring scheme for the small coal pillar. Each set of measurement stations was equipped with four borehole stress meters, each with lengths of 1 m, 2 m, 3 m, and 4 m and with a spacing of 0.5 m between them. Changes in borehole stress readings at various distances from the working face were monitored during the retreat of the 6212 mining panel.

**Fig. 18:**
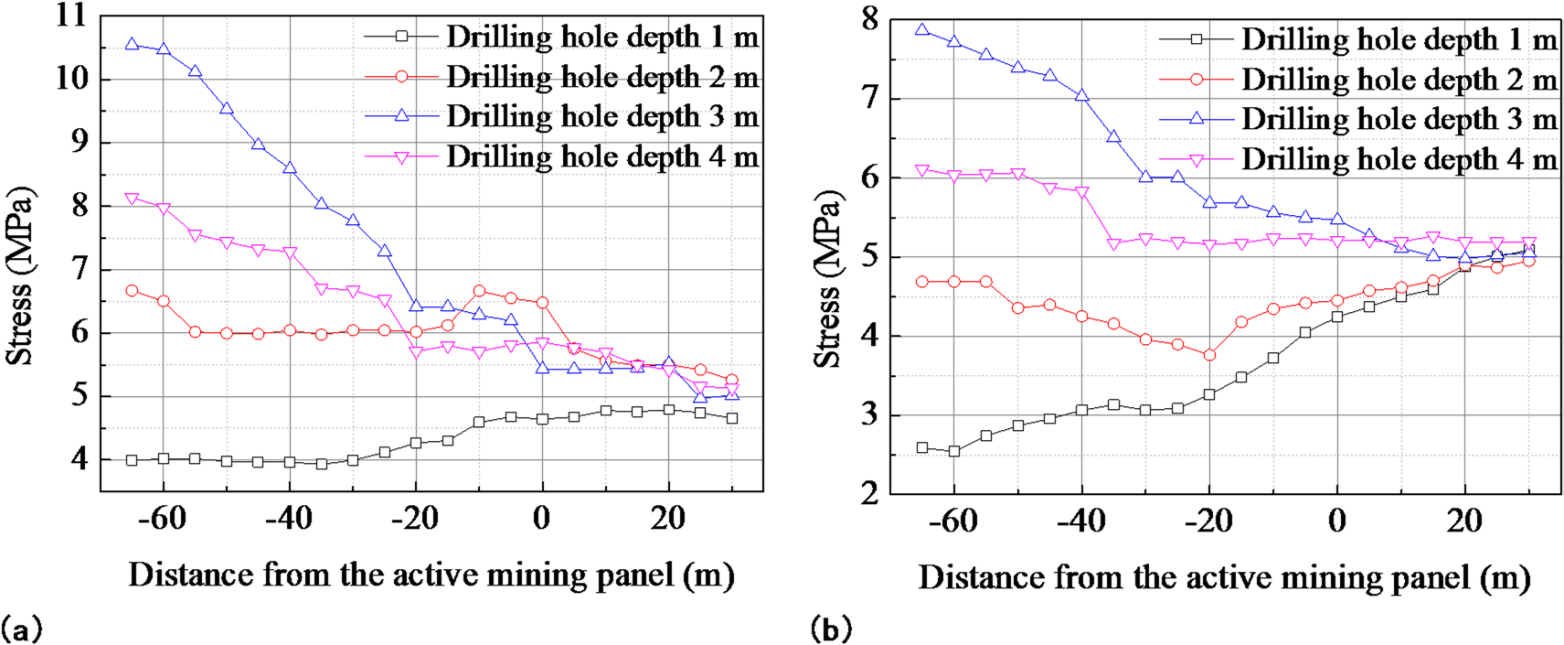
Borehole stress change curve of small coal pillar: **(a)** no prefabricated cracks relief section; **(b)** prefabricated cracks relief section.

The pressure change curve of the small coal pillar is shown in Fig. 18. There was less stress in the prefabricated crack relief section than in the non-relief section at the same drilling depth. The increase in stress in the unrelieved section was 5.5 MPa and 3.0 MPa at depths of 3 and 4 m, respectively. In contrast, the stress increase in the prefabricated crack relief section was 2.5 MPa and 1.0 MPa, respectively, indicating that the stress increased less in this section than in the unrelieved one. The borehole stress monitors installed at depths of 3 m and 4 m are positioned 0.5 m and 1.5 m from the center of the small coal pillar, respectively. The difference in the measured stresses is thus primarily attributable to variations in the stress distribution at different locations within the small coal pillar. After adopting deep-hole directional prefabricated cracks on the roof, there was a reduction in stress for the small coal pillar, which contributed to its maintenance.

## Conclusions

This study takes a small coal pillar roadway as the research object. Through numerical simulation, physical similarity simulation, and field verification, the stress superposition characteristics, energy accumulation law, and surrounding rock deformation behavior of small coal pillar roadway under multi-time dynamic disturbances are systematically revealed. The improvement mechanism of prefabricated roof cracks on overburden caving and strata structure is clarified, and the field pressure relief effect is verified. The main conclusions are as follows:

1. Under the superposition of four dynamic disturbance, the vertical stress peaks on the small coal pillar gradually increase to 12.96 MPa, 14.30 MPa, 22.11 MPa, and 24.49 MPa, respectively. The continuous superposition of dynamic stresses leads to severe stress concentration in the small coal pillar, which significantly deteriorates its stability.
2. After the excavation of the two adjacent roadways, the elastic strain energy density at the coal pillar center reaches 59.93 kJ/m³. During panel retreat, the strain energy density at 30 m behind the mining panel increases by 138.72%. The continuous mining operation further aggravates energy accumulation, which is the key factor inducing the instability and failure of small coal pillar.
3. The prefabricated roof cracks effectively optimize the overburden caving structure. The roof strata cave along the prefabricated cracks, and the caving angle increases to approximately 70.5°. Meanwhile, the long cantilever structure is transformed into a short cantilever, with the overhang length reduced by 48%. The prefabricated cracks significantly increase the caving angle and shorten the overhang length, thus fundamentally improving the roof stress environment.
4. Field application shows that prefabricated crack pressure relief presents an obvious control effect. Before pressure relief, the stress increments at borehole depths of 3 m and 4 m are 5.5 MPa and 3.0 MPa, respectively; after pressure relief, they decrease to 2.5 MPa and 1.0 MPa. The results verify that the prefabricated crack technology can effectively reduce stress concentration and ensure the stability of small coal pillar roadways.

## Data Availability

The original contributions presented in the study are included in the article, further inquiries can be directed to the corresponding author.
